# INTEGRATING HEALTH AND HOME CARE THROUGH TECHNOLOGY: ACTIONABLE SOLUTIONS FOR PROGRESS

**DOI:** 10.1093/geroni/igac059.928

**Published:** 2022-12-20

**Authors:** Lauren Dunning, Caroline Servat, Nora Super

**Affiliations:** Milken Institute Center for the Future of Aging, Santa Monica, California, United States; Milken Institute Center for the Future of Aging, Santa Monica, California, United States; Milken Institute Center for the Future of Aging, Santa Monica, California, United States

## Abstract

Shifts toward virtual care driven by the pandemic have continued to blur the previously sharp lines between health care and home care. A new care ecosystem is emerging, bringing with it the opportunity to integrate health and home care through technology. Over the course of the pandemic, Medicare telehealth visits increased 63-fold between 2019 and 2020, innovative programs enabled acute hospital care at home using digital tools, and people in need of care could reach their providers through a broad range of devices and modalities. But many of the policy changes underlying these advancements and others were temporary, and permanent actions are needed to sustain momentum. To characterize the emerging landscape, the Milken Institute conducted informational interviews with over 40 experts representing health, technology, government and policy, research and academia, philanthropy, advocacy, and community-based organizations. From these interviews, themes emerged on barriers, innovations, and opportunities. Findings were further refined through a roundtable workshop and survey to develop guiding principles and consensus-built recommendations. Three sets of recommendations were then developed on: 1) pandemic-related and larger-scale policy and program design to support tech-enabled care, 2) practices and policies to develop a systems approach that integrates health and home care and bolsters equity, and 3) collaboration and coordination to accelerate efforts. This paper focuses on the interview themes and progress made to advance solutions in partnership with stakeholders.

